# Methodological aspects of the highly adaptive testing design for PISA

**DOI:** 10.3389/fpsyg.2024.1446799

**Published:** 2024-09-17

**Authors:** Aron Fink, Christoph König, Andreas Frey

**Affiliations:** Institute of Psychology, Goethe University Frankfurt, Frankfurt, Germany

**Keywords:** computerized adaptive testing, PISA, testing, measurement, item response theory

## Abstract

This methods paper describes the methodological and statistical underpinnings of the highly adaptive testing design (HAT), which was developed for the Programme for International Student Assessment (PISA). The aim of HAT is to allow for a maximum of adaptivity in selecting items while taking the constraints of PISA into account with appropriate computer algorithms. HAT combines established methods from the area of computerized adaptive testing (a) to improve item selection when items are nested in units, (b) to make use of the correlation between the dimensions measured, (c) to efficiently accomplish constraint management, (d) to control for item position effects, and (e) to foster students’ test-taking experience. The algorithm is implemented using the programming language R and readers are provided with the necessary code. This should facilitate future implementations of the HAT design and inspire other adaptive testing designs that aim to maximize adaptivity while meeting constraints.

## 1 Introduction

In recent years, the Programme for International Student Assessment (PISA) has made considerable changes to its assessment design. The main goal of these changes was to increase the accuracy with which student proficiency is measured. In the 2018 assessment cycle, PISA moved from fixed test forms to multistage adaptive testing for the reading domain (MST; Yamamoto et al., 2019). In 2022, MST was introduced for the mathematics domain (OECD, in press). MST strives for a more individualized item assignment to achieve a better fit between the difficulty of the presented items and the individual proficiency level of the students, thereby preventing the use of items that are far too easy or far too difficult. An adaptive item selection increases the precision of individual student ability estimations, especially in low- and high-achieving countries. Thus, it makes it possible to match the level of precision that was previously only reached in countries with an average performance. By making it possible to measure across a broader ability range, a more diverse group of students can be measured, thereby extending the global reach of PISA.

The introduction of MST resulted in an increase of 4–7% in terms of test information (Yamamoto et al., 2019). This increase can be characterized as modest. When looking into the literature on computerized adaptive testing (CAT; e.g., Frey, 2023), the PISA 2018 MST design is more restrictive than necessary and larger increases in test information would have been expected with a higher degree of adaptivity. However, fully unconstrained adaptive test designs are not feasible for PISA, which has numerous constraints besides statistical optimality. Amongst other constraints, adaptive test designs have to take into account that most items are nested in units (testlets) in PISA and that PISA’s assessment framework includes cognitive processes and domain-specific substructures, as well as the use of link items. Empirical evidence, however, highlights that a considerable increase in test information is possible while taking a wide range of different constraints into account (Frey and Seitz, 2011 and Frey et al., 2013 for PISA; Frey and Fink, 2021 for the Teaching and Learning International Survey, TALIS).

The purpose of this methods paper is to outline and formalize the algorithm of the highly adaptive testing design (HAT; Frey et al., 2023) for PISA, which is based on CAT with shadow testing (e.g., van der Linden and Reese, 1998). The HAT design for PISA maximizes the adaptivity while taking the core constraints of PISA assessments into account, thus making it feasible for application in operational PISA settings. The HAT design for PISA combines methods (a) to improve item selection when items are nested in units, (b) to make use of the correlation between the dimensions measured, (c) to efficiently accomplish constraint management, (d) to control for item position effects, and (e) to foster students’ test-taking experience. In the following, the core elements of the HAT design for PISA are described in detail.

## 2 Elements of the highly adaptive testing design

### 2.1 Unit-level item selection with within-unit adaptation

The operational item pool of PISA consists of dichotomous and polytomous items. The HAT design for PISA accounts for this mixture of item types by utilizing the multidimensional two-parameter logistic item response theory (IRT) model (M2PL; e.g., Reckase, 2009) for the former and the generalized partial credit model (GPCM; Muraki, 1992) for the latter item type. This is in line with the scaling practice currently adopted in PISA (OECD, 2024). Under the M2PL and assuming *D* dimensions, the probability of person j to correctly answer item *i*, *i* = 1,…, *I* is denoted as $p_{i}\left(y_{ji}=1|\theta_{j},a_{i},b_{i}\right)=\mathrm{logit}\left[a_{i}^{\prime }\left(\theta_{j}-b_{i}1\right)\right]$ , where $\theta_{j}$ is the D×1 ability vector of person j , $a_{i}$ is the D×1 vector of discriminations for item i , and $b_{i}$ is the difficulty of item *i,* which is multiplied by a D×1 vector of ones so that the difficulty is applied to all dimensions (e.g., Segall, 1996). The majority of operational PISA items are organized into units that share a common stimulus. While unit-level selection offers fewer routing options compared to item-level CAT, it still provides more routing possibilities than the two routings incorporated into the PISA 2018 MST design, thus resulting in a more nuanced adaptation. Due to the construction of units, which aims to make the mean item difficulty comparable across units, choosing entire units would allow for a reduced amount of adaptivity only. The HAT design for PISA utilizes within-unit adaptivity to select only the items with the highest information from the selected units. The maximum number of units and the maximum number of items per unit are specified as constraints using the shadow-test approach (see Constraint Management section).

### 2.2 Making use of the correlation between measured dimensions

In the HAT design for PISA, additional information, which comes from the covariances between the domains of reading, mathematics, and science, is used for multidimensional ability estimation. In addition, the HAT design for PISA adopts the principles of multidimensional adaptive testing (MAT; e.g., Frey and Seitz, 2009) to further refine the unit and item selection process. An unrestricted MAT algorithm is likely to present frequent changes in the domains measured, which may distract the test takers. In order to avoid the possibility of such distractions, the HAT design for PISA follows the operational PISA practice of presenting sequences of units (“clusters”) that all measure the same domain. The complete proficiency test consists of four 30-min clusters. The HAT design for PISA covers two clusters for the major domain of PISA 2018, which was reading (60 min of testing time), followed by two adaptive clusters from one of the two minor domains, which were mathematics and science (also 60 min of testing time). This resulted in 24 different test forms with varying combinations of major and minor domains (see Table 1). The test forms are supposed to be randomly assigned to students (e.g., by spiraling within classrooms). Within each test form, the item administration was unidimensional. HAT, however, still makes it possible to utilize the information about the high correlation between the reading, mathematics, and science domains by setting the initial ability level $\hat{\theta }$ of the first cluster of the second domain (Cluster 3) to the final $\hat{\theta }$ of the second cluster of the first domain (Cluster 2).

**Table 1:** The adaptive test forms of the HAT design for PISA 2018.

Form	Cluster 1	Cluster 2	Cluster 3	Cluster 4
1	READ1	READ2	MATH1	MATH2
2	READ2	READ1	MATH2	MATH1
3	READ1	READ2	MATH2	MATH1
4	READ2	READ1	MATH1	MATH2
5	MATH1	MATH2	READ1	READ2
6	MATH2	MATH1	READ2	READ1
7	MATH1	MATH2	READ2	READ1
8	MATH2	MATH1	READ1	READ2
9	SCIE1	SCIE2	READ1	READ2
10	SCIE2	SCIE1	READ2	READ1
11	SCIE1	SCIE2	READ2	READ1
12	SCIE2	SCIE1	READ1	READ2
13	READ1	READ2	SCIE1	SCIE2
14	READ2	READ1	SCIE2	SCIE1
15	READ1	READ2	SCIE2	SCIE1
16	READ2	READ1	SCIE1	SCIE2
17	MATH1	MATH2	SCIE1	SCIE2
18	MATH2	MATH1	SCIE2	SCIE1
19	MATH1	MATH2	SCIE2	SCIE1
20	MATH2	MATH1	SCIE1	SCIE2
21	SCIE1	SCIE2	MATH1	MATH2
22	SCIE2	SCIE1	MATH2	MATH1
23	SCIE1	SCIE2	MATH2	MATH1
24	SCIE2	SCIE1	MATH1	MATH2

READ, Reading domain; MATH, Mathematics domain; SCIE, Science domain.

### 2.3 Constraint management

#### 2.3.1 Shadow-test approach

In addition to selecting highly informative items within units, the HAT design for PISA uses the shadow-test approach to account for the numerous constraints of PISA. Satisfying these constraints is crucial for the derivation of valid test score interpretations. The different constraints of the HAT design for PISA 2018 (reading domain) are presented in Table 2. The constraints regarding the three cognitive domains were derived from the PISA 2018 assessment and analytical framework for reading (OECD, 2019). Please note that the HAT design for PISA allows for a 1:1 translation of the assessment and analytical framework for the three cognitive domains of reading, mathematics, and science into constraints for the shadow test. The constraints for mathematics and science are illustrated in Tables 3, 4. The HAT design for PISA implements a shadow test model that optimally selects the *k*th item while preserving the desired values of these parameters (for a detailed description of the shadow-test approach, see van der Linden, 2022).

**Table 2:** Constraints for the PISA 2018 reading HAT design.

ID	Constraint	LB	UB
1	Number of items per test (test length)	36	36
2	Number of units per test	12	12
3	Number of items per unit	3	3
4	Number of items of cognitive process “Scan”	4	6
5	Number of items of cognitive process “Represent”	4	6
6	Number of items of cognitive process “Integrate” (Single text)	4	6
7	Number of items of cognitive process “Integrate” (Multiple texts)	4	6
8	Number of items of cognitive processes “Reflect” and “Assess”	6	8
9	Number of items of cognitive process “Corroborate”	3	5
10	Number of items of cognitive process “Search”	3	5
11	Number of human-coded items	7	17
12	Number of single-text items	24	35
13	Number of multiple-text items	1	13
14	Number of trend items	3	21

LB, lower bound; UB, upper bound.

**Table 3:** Constraints for the PISA 2018 mathematics HAT design.

ID	Constraint	LB	UB
1	Number of items per test (test length)	24	24
2	Number of units per test	10	10
3	Number of items per unit	1	4
4	Number of items of cognitive process “Employ”	7	9
5	Number of items of cognitive process “Formulate”	7	9
6	Number of items of cognitive process “Interpret”	7	9
7	Number of items of content category “Change”	5	7
8	Number of items of content category “Space”	5	7
9	Number of items of content category “Quantity”	5	7
10	Number of items of content category “Uncertainty”	5	7
11	Number of human-coded items	4	8
12	Number of machine-coded items	16	18

LB, lower bound; UB, upper bound.

**Table 4:** Constraints for the PISA 2018 science HAT design.

ID	Constraint	LB	UB
1	Number of items per test (test length)	36	36
2	Number of units per test	12	12
3	Number of items per unit	1	5
4	Number of items of competency “Evaluate”	7	11
5	Number of items of competency “Explain”	14	18
6	Number of items of competency “Interpret”	11	14
7	Number of items of knowledge “Content”	19	24
8	Number of items of knowledge “Epistemic”	4	8
9	Number of items of knowledge “Procedural”	7	11
10	Number of items of system “Earth”	9	11
11	Number of items of system “Living”	12	14
12	Number of items of system “Physical”	12	14
13	Number of human-coded items	6	14
14	Number of machine-coded items	23	33
15	Number of trend items of type “Standard”	26	35
16	Number of trend items of type “Interactive”	5	10

LB, lower bound; UB, upper bound.

To assemble a test of *t* items from a calibrated pool of *I* items ( i=1,…,I ) that provides maximum information at point $\theta_{k}$ of the measured scale, the first step is to identify the decision variables for each possible solution. This can be done by using *I* binary variables ( $x_{1}$ , … , $x_{I}$ ), where $x_{i}=1$ if item *i* is selected and 0 otherwise. Each combination of values for these variables represents a different test. The Fisher information for each item *i* at $\theta_{k}$ is represented as $I_{i}\left(\theta_{k}\right)$ ; under the 2PL IRT model (because item selection in the HAT design for PISA is unidimensional), the Fisher information is calculated as $I_{i}\left(\theta_{k}\right)=a_{i}^{2}P_{i}\left(\theta_{k}\right)Q_{i}\left(\theta_{k}\right)$ , where $P_{i}\left(\theta_{k}\right)=\mathrm{logit}\left[a_{i}\left(\theta_{k}-b_{i}\right)\right]$ and $Q\left(\theta_{k}\right)=1-P_{i}\left(\theta_{k}\right)$ . The objective function for automated test assembly can be written as


$$\mathrm{maximize}\;\overset{I}{\underset{i=1}{\sum }}I_{i}\left(\theta_{k}\right)x_{i}\text{.}$$

For the PISA 2018 HAT design for reading, for example, the first constraint is the test length *t*. We used a test length of 36 items. The corresponding constraint can be formulated as


$$\overset{I}{\underset{i=1}{\sum }}x_{i}=36.$$

Additionally, items frequently fall into various subsets $V_{c}$ , where $i\in V_{c};c=1,\ldots ,C\text{.}$ For instance, a subset may exclusively contain items that belong to a particular content category (e.g., items belonging to the cognitive process “represent”) and a special item type (e.g., machine- vs. human-coded items). To account for this, categorical constraints can be incorporated into the model, such as specifying the desired number of items $n_{c}$ from the subset $V_{c}$ as


$$\underset{i\in V_{c}}{\sum }x_{i}⋚n_{c},c=1,\ldots C\text{,}$$

where ⋚ denotes the choice of a (strict) (in)equality. All the constraints of PISA that are incorporated into the HAT design can be expressed in this way. For example, the number of items in each individual test from the subset of items $V_{\mathrm{Rep}}$ measuring the cognitive process “represent” should be between four and six. The respective constraint can be formulated as follows:


$$\underset{i\in V_{\mathrm{Rep}}}{\sum }x_{i}\geq 4\text{,}$$

$$\underset{i\in V_{\mathrm{Rep}}}{\sum }x_{i}\leq 6.$$

As most operational PISA items are grouped into units, an extra set of binary decision variables is needed for the level of units. Let s=1,…,S denote each stimulus in an item pool and $z_{1},\ldots ,z_{S}$ its corresponding decision variables so that $z_{S}=1$ if stimulus s is selected and 0 otherwise. The existence of a hierarchical structure between the items and the stimuli requires the inclusion of additional logical constraints. For instance, selecting an item that belongs to a particular unit necessitates the selection of the stimulus as well. Therefore, the following condition needs to be met:


$$x_{i_{s}}-z_{s}\leq 0\text{,}$$

where $i_{s}\in V_{s}$ denotes the items belonging to unit *s.* In addition, the minimum/maximum number of items $n_{s}$ to be selected per unit can be specified via the constraint


$$\underset{i_{s}\in V_{s}}{\sum }x_{i_{s}}⋚n_{s}z_{s}\text{.}$$

This constraint ensures that the constraint is only triggered when $z_{s}=1\text{,}$ which means that the respective unit was selected. In addition to these kinds of constraints, there are numerous other possibilities (see van der Linden, 2022 for an overview of how to formulate different kinds of constraints in the shadow-test approach).

In adaptive item selection, the automated test assembly procedure described above, with all the constraints, has to be accomplished after each update of the provisional ability estimate. The general idea is to select items from a hypothetical test (i.e., a shadow test), which is compiled automatically before the selection of each item, instead of selecting from the complete item pool. The algorithm can be described as follows:

1. Initialize the ability estimation.
2. Assemble a shadow test that accounts for all constraints (e.g., test length, content coverage, number of units, number of items per unit, etc.), contains all items already administered, and provides maximum Fisher information at the current provisional ability estimate.
3. Administer an item from the shadow test that was not yet administered and that has maximum Fisher information at the current ability estimation. Return all unused items to the item pool.
4. Update the ability estimate.
5. Update the constraints to consider the attributes of the items already administered.
6. Repeat Steps 1–5 until the termination criterion of the adaptive test is met.

To automatically include all items in the set $S_{k-1}$ that have already been administered in the next shadow test (Step 5), the following constraint has to be added:


$$\underset{i\in S_{k-1}}{\sum }x_{i}=k-1.$$

Assembling each shadow test at each step to satisfy all constraints imposed ensures that the resulting set of presented items satisfies all the constraints. Furthermore, the shadow tests are assembled to provide maximum information with regard to the provisional ability estimate at each step. Normally, the shadow test has to be assembled in real time before the administration of each item. This process is handled by an automated test assembly method that uses mixed-integer programming. Solvers for this linear type of optimization are available in different software packages (see Technical Implementation section).

#### 2.3.2 Item exposure

Allowing an unrestricted adaptive unit and item selection could result in uneven item exposure rates, with some items being presented more frequently than others. This can cause problems in calibration and also regarding content coverage. The HAT design for PISA therefore utilizes Revuelta and Ponsoda’s (1998) progressive item exposure method to control the item exposure rates. The progressive method adds a random component to the maximum Fisher information item selection criterion. The influence of this random component gets smaller over the course of the test. It is most influential at the beginning of the test, where the provisional ability estimate is less precise. As the test progresses, the provisional ability estimate becomes more precise, and the item selection is based more on the item information. The algorithm of the progressive method is as follows (Revuelta and Ponsoda, 1998):

1. Calculate the Fisher information $I_{i}$ for each unused item i , based on the ability estimate ${\hat{\theta }}_{j}$ of student *j* obtained from the *h* items already administered. The highest information value obtained is denoted as *H.*
2. For each unused item i , draw a random value $R_{i}$ from a uniform distribution (0, *H*).
3. Compute a weight $w_{i}$ for each unused item i as a linear combination of the random value $R_{i}$ and the information $I_{i}$ by $w_{i}=\left(1-s\right)R_{i}+sI_{i}$ , where s equals the relative serial position of the item in a test with test length t , calculated by s=h/t .
4. Use this weight instead of the item information in the item selection procedure.

Another relevant aspect is that the PISA main study data is also used to estimate item parameters and to link the scale of the current assessment to the existing reporting scale. This necessitates that the numbers of responses for the individual items do not fall below a minimum number. This minimum threshold can be assured by incorporating an additional constraint: in specific test positions, items are not chosen adaptively, but rather in a fixed spiraling sequence. Therefore, in the HAT design for PISA, each student received two complete units during the reading test. These non-adaptive, complete units were administered in a spiraling fashion such that Student 1 received Unit 1 and Unit 2, Student 2 received Units 2 and 3, Student 4 received Units 3 and 4, and so on. In this way it is ensured, that each item has a minimum number of 250 responses in each participating country.

### 2.4 Item position effects

Item position effects (IPEs; e.g., Nagy et al., 2019) refer to situations in which the properties of items vary depending on their position in a test. A typical case of an IPE is an increase in item difficulty estimates towards the end of a test. Especially for CAT, IPEs pose a significant challenge because, in CAT, the item parameter estimates are fixed during item selection and ability estimation. Moreover, in CAT, items are often presented more frequently in specific positions than in others. Disregarding IPEs in CAT can result in substantial bias, as emphasized and demonstrated by Frey and Fink (in press). This problem can be avoided by balancing the positions in which items are presented. This was incorporated into the HAT design for PISA by introducing the principle of the continuous calibration strategy (Fink et al., 2018; Frey and Fink, in press) simply as an additional constraint in the shadow-test approach. By means of automated test assembly, the domain-specific item pool was divided into two subpools of equal length (18 items), ensuring that each subpool complied with all imposed constraints. The allocation of items to subpools was then introduced twice as an additional item attribute (once for each cluster within a domain), but in reversed order. The IPE constraint specified that, within a domain, items from both subpools were to be administered in each cluster, taking the reverse order into account. For example, in the first cluster of the first domain, items from the first subpool were to be administered prior to items from the second subpool, while, in the following second cluster of the same domain, items from the second subpool were to be administered prior to items from the first subpool. This constraint balances item positions on the level of clusters.

### 2.5 Students’ test-taking experience

In order to foster students’ test-taking experience, the possibility of increasing the desired average response probability that is used for the adaptive item selection is implemented into the HAT design for PISA. According to the meta-analysis of Frey et al., 2024, this can be expected to reduce negative test taker emotions such as anxiety and anger. For this purpose, the HAT design for PISA utilizes the procedure outlined by Eggen and Verschoor (2006). The goal is to select easier items with higher success probabilities compared to items that are selected with maximum Fisher information. Therefore, the item selection is based on the computation of the Fisher information $I\left(\hat{\theta }-\delta_{i}\right)$ at an ability level that is shifted away from the current ability estimate $\hat{\theta }$ by $\delta_{i}$ . For the 2PL model, this shift can be calculated by $\delta_{i}=\frac{1}{a_{i}}\ln\left[\frac{p_{i}\left(\theta -\delta_{i}\right)}{1-p_{i}\left(\theta -\delta_{i}\right)}\right]$ , where $p_{i}\left(\theta -\delta_{i}\right)$ equals the desired response probability. On the basis of the way in which the proficiency levels are defined in PISA, we suggest using a response probability (RP) of .62 for a correct answer when calculating the maximum Fisher information during HAT item selection. Therefore, the item is selected with maximum Fisher information at $\theta =\hat{\theta }-\delta_{i}=\hat{\theta }-\frac{1}{a_{i}}\ln\left(\frac{.62}{.38}\right)$ . Please note that the HAT design for PISA is not restricted to an RP of .62 and can be used with other RP values too.

### 2.6 Adaptive algorithm

**Figure 1:**
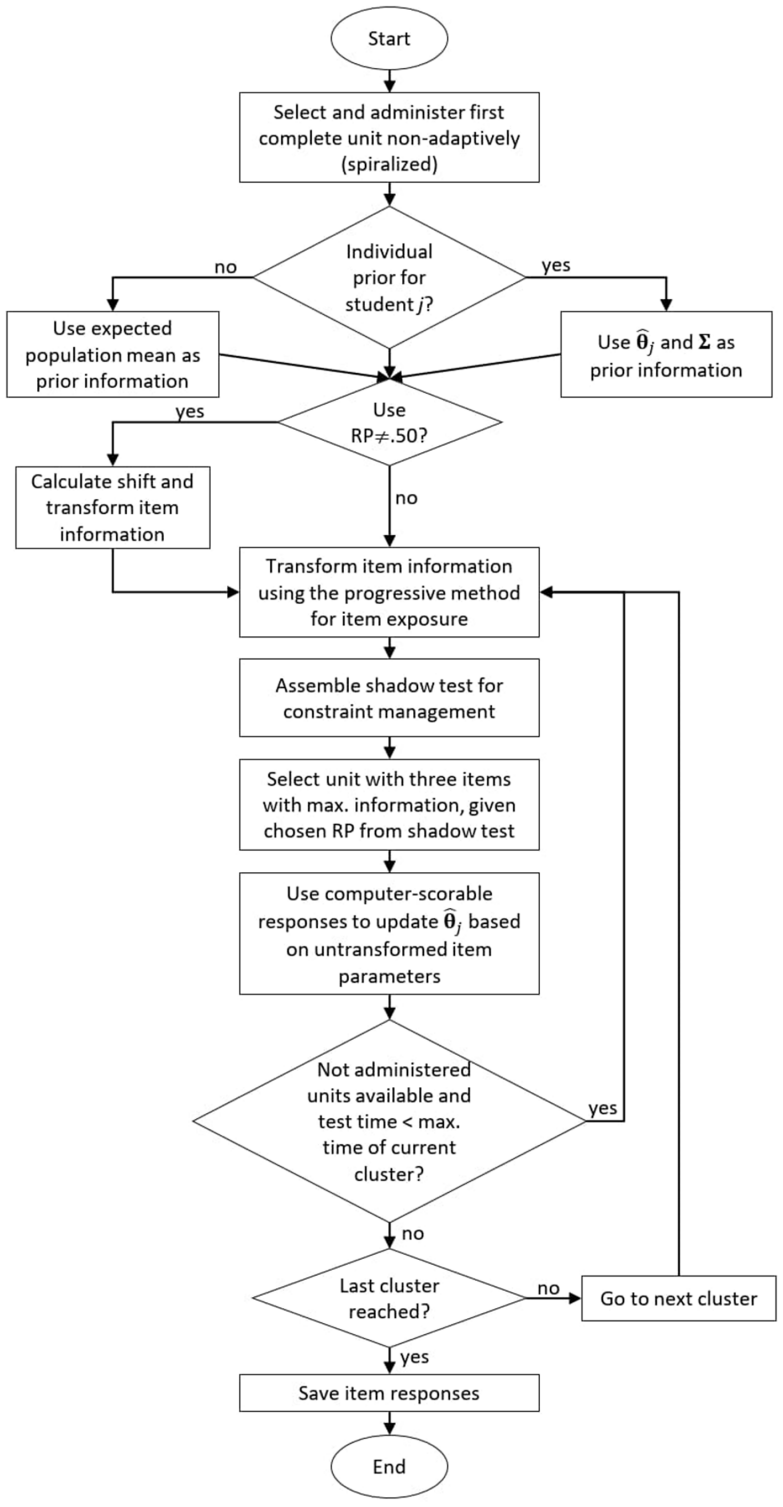
Flow chart of the highly adaptive testing design for PISA. Note: ${\hat{\theta }}_{j}$ = provisional abibilty estimate of person *j*, Σ = covariance matrix of the *D* latent dimensions, RP = response probability.

The complete algorithm of the HAT design for PISA is summarized by the flow chart in Figure 1.

### 2.7 Technical implementation

Each element of the algorithm of the HAT design for PISA can be easily implemented in open-source statistical software. Its code is transparent and adaptable and can be accessed here: https://doi.org/10.17605/OSF.IO/RV5YX. The current version of the HAT design for PISA runs in the R environment (R Core Team, 2022) and uses only two packages, namely, a modified version of the *TestDesign* package (Choi et al., 2022) for test administration and shadow testing, and *mirt* (Chalmers, 2012) for IRT scaling. For linear optimization, the current version of the HAT design for PISA draws on the *gurobi* solver (Version 10.0; Gurobi Optimization, LLC, 2023). Please note that the HAT design for PISA is not bound to this optimizer. For operational applications of the HAT design for PISA, open-source optimizers such as the R packages *lpSolve* (Berkelaar et al., 2022) and *RSymphony* (Harter et al., 2021) can also be used.

## 3 Concluding remarks

This methods paper describes the methodological and statistical underpinnings of the HAT design for PISA. This design maximizes the amount of adaptivity given typical PISA constraints, making it feasible for operational PISA assessments. We showed that the implementation of the core algorithm and its elements is actually quite simple. The code necessary to use the HAT design is transparent, parsimonious, and manageable. We see this as an important step towards increasing the transparency of the PISA methodology, which was considered by Frey and Hartig (2020) to be one of the five current methodological challenges of international large-scale assessments. Using linear optimization renders the manual allocation of items, units, and stages obsolete, resulting in a better and more in-depth representation of the PISA assessment framework in the actual assessment. The implementation is flexible and can accommodate potential changes in assessment frameworks or the operational procedures of the assessments. Lastly, the HAT design for PISA can be implemented with open-source software solutions; proprietary software is not necessary.

To back up these arguments with empirical evidence, the performance of the HAT design for PISA in terms of relative efficiency, accuracy, exposure control, and constraint violations was investigated in a comprehensive simulation study (Frey et al., 2023). Compared to the PISA 2018 MST reading design, the HAT design for PISA resulted in an increase in test information of approximately 30% across a range of ability groups. Ability estimates obtained with the HAT design for PISA were more accurate (in terms of the RMSE) than those obtained with the MST design. Moreover, the HAT design for PISA had clear advantages over the MST design regarding constraint violations: while the latter violated approximately four constraints on average, the former violated none. Thus, with its shadow-test approach, the HAT design for PISA indeed maximizes test information in the context of a relatively large number of potentially conflicting constraints. An area for possible improvement is, however, exposure control. Here, the MST design showed a more uniform administration of items, compared to that of the HAT design for PISA. Items in the HAT design for PISA, however, showed exposure rates that are entirely feasible for operational use. One way to optimize the item exposure of HAT is to systematically expand the item pool. Currently, a number of low-discriminating and very difficult items prevent a uniform item usage when the maximum Fisher information criterion is used. A targeted development of the item pool, including an examination of whether items with low *a*-parameters can be excluded, would not only affect exposure rates positively; it would also increase the gains in test information. Frey et al. (2023) were able to show that, in the case of an optimal item pool, the test information increased almost threefold compared to the PISA 2018 MST reading design.

The gain in test information associated with the HAT design for PISA is considerable. It is likely that the reduction of the measurement error due to this gain positively affects the precision of the population estimates used for PISA reporting. Thus, more fine-grained results and/or more power for statistical tests can be expected.

We hope that this methods paper is useful for future implementations of the HAT design or that it inspires other testing designs that strive to maximize adaptivity while meeting constraints.

## Data Availability

The original contributions presented in the study are included in the article/supplementary material, further inquiries can be directed to the corresponding author.

## References

[ref9001] Berkelaar M. . (2022). lpSolve: Interface to ‘Lp_solve’ v. 5.5 to Solve Linear/Integer Programs. R package version 5.6.17. https://CRAN.R-project.org/package=lpSolve

[ref1] Chalmers R. P. (2012). mirt: a multidimensional item response theory package for the R environment. J. Stat. Softw. 48, 1–29. doi: 10.18637/jss.v048.i06

[ref2] Choi S. W. Lim S. van der Linden W. (2022). TestDesign: an optimal test design approach to constructing fixed and adaptive tests in R. Behaviormetrika 49, 191–229. doi: 10.1007/s41237-021-00145-9

[ref3] Eggen T. J. H. M. Verschoor A. J. (2006). Optimal testing with easy or difficult items in computerized adaptive testing. Appl. Psychol. Meas. 30, 379–393. doi: 10.1177/0146621606288890, PMID: 39176014

[ref4] Fink A. Born S. Spoden C. Frey A. (2018). A continuous calibration strategy for computerized adaptive testing. Psychol. Test Assess. Model. 60, 327–346.

[ref9002] Frey A. (2023). Computerized adaptive testing and multistage testing. In International Encyclopedia of Education, (eds.) Tierney R. J. Rizvi F. Erkican K.. vol. 14 4th Edition, (Amsterdam: Elsevier), pp. 209–216.

[ref9003] Frey A. Fink A. (2021). Increasing test efficiency in an international assessment of teachers’ general pedagogical knowledge through multidimensional adaptive testing. In Teaching as a knowledge profession. Studying pedagogical knowledge across educational systems. (ed.) Ulferts H. (Paris: OECD Publishing), pp. 123–140.

[ref5] Frey A. Fink A. (in press). Controlling for item position effects when adaptive testing is used in large-scale assessments. In Khorramdel L. von Davier M. Yamamoto K. (Eds.), Innovative computer-based international large-scale assessments – Foundations, methodologies, and quality assurance procedures. Cham: Springer.

[ref6] Frey A. Hartig J. (2020). “Methodological challenges of international student assessment” in Monitoring of student achievement in the 21st century. eds. Harju-Luukkainen H. McElvany N. Stang J. (Cham: Springer), 39–49.

[ref7] Frey A. König C. Fink A. (2023). A highly adaptive testing design for PISA. J. Educ. Meas. doi: 10.1111/jedm.12382 PMC11442237 39355299

[ref8] Frey A. Liu T. Fink A. König C. (2024). Meta-analysis of the effects of computerized adaptive testing on the motivation and emotion of examinees. Eur. J. Psychol. Assess. doi: 10.1027/1015-5759/a000821

[ref9] Frey A. Seitz N. N. (2009). Multidimensional adaptive testing in educational and psychological measurement: current state and future challenges. Stud. Educ. Eval. 35, 89–94. doi: 10.1016/j.stueduc.2009.10.007

[ref10] Frey A. Seitz N. N. (2011). Hypothetical use of multidimensional adaptive testing for the assessment of student achievement in PISA. Educ. Psychol. Meas. 71, 503–522. doi: 10.1177/0013164410381521

[ref9004] Frey A. Seitz N. N. Kröhne U. (2013). Reporting differentiated literacy results in PISA by using multidimensional adaptive testing. In Research on PISA. (eds.) Prenzel M. Kobarg M. Schöps K. Rönnebeck S. (Dordrecht: Springer), pp. 103–120.

[ref11] Gurobi Optimization, LLC (2023). Gurobi optimizer reference manual. Available at: https://www.gurobi.com (Accessed June 10, 2023).

[ref12] Harter R. Hornik K. Theussl S. (2021). Rsymphony: SYMPHONY in R. R package version 0.1-33. Available at: https://CRAN.R-project.org/package=Rsymphony (Accessed June 10, 2023).

[ref9005] Muraki E. (1992). A generalited partial credit model: application of an em algorithm. ETS Research Report Series, 1–30. doi: 10.1002/j.2333-8504.1992.tb01436.x

[ref13] Nagy G. Nagengast B. Frey A. Becker M. Rose N. (2019). A multilevel study of position effects in PISA achievement tests: student- and school-level predictors in the German tracked school system. Assess. Educ. Prin. Policy Pract. 26, 422–443. doi: 10.1080/0969594X.2018.1449100

[ref14] OECD (2019). PISA 2018 assessment and analytical framework. Paris: OECD Publishing.

[ref15] OECD (2024). PISA 2022 technical report. Paris: OECD Publishing.

[ref16] OECD (in press). PISA 2018 technical report. Paris: OECD Publishing.

[ref17] R Core Team (2022). R: A language and environment for statistical computing [software]. Vienna: R Foundation for Statistical Computing.

[ref18] Reckase M. D. (2009). Multidimensional item response theory. New York: Springer.

[ref19] Revuelta J. Ponsoda V. (1998). A comparison of item exposure control methods in computerized adaptive testing. J. Educ. Meas. 35, 311–327. doi: 10.1111/j.1745-3984.1998.tb00541.x, PMID: 35601264

[ref20] Segall D. O. (1996). Multidimensional adaptive testing. Psychometrika 61, 331–354. doi: 10.1007/BF02294343, PMID: 39167457

[ref21] van der Linden W. J. (2022). Review of the shadow-test approach to adaptive testing. Behaviormetrika 49, 169–190. doi: 10.1007/s41237-021-00150-y, PMID: 29575849

[ref22] van der Linden W. J. Reese L. M. (1998). A model for optimal constrained adaptive testing. Appl. Psychol. Meas. 22, 259–270. doi: 10.1177/01466216980223006

[ref9006] Yamamoto K. Shin Khorramdel L. (2019). Introduction of multistage adaptive testing design in PISA 2018. OECD Education Working Papers, No. 209. Paris: OECD Publishing.

